# A new function for the serine protease HtrA2 in controlling radiation‐induced senescence in cancer cells

**DOI:** 10.1002/1878-0261.13187

**Published:** 2022-02-16

**Authors:** Liat Hammer, Vered Levin‐Salomon, Naama Yaeli‐Slonim, Moria Weiss, Naama P. Dekel‐Bird, Tsviya Olender, Ziv Porat, Sabina Winograd‐Katz, Alon Savidor, Yishai Levin, Shani Bialik, Benjamin Geiger, Adi Kimchi

**Affiliations:** ^1^ 34976 Department of Molecular Genetics Weizmann Institute of Science Rehovot Israel; ^2^ 34976 Department of Life Sciences Core Facilities Weizmann Institute of Science Rehovot Israel; ^3^ 34976 Department of Immunology Weizmann Institute of Science Rehovot Israel; ^4^ 34976 The Nancy and Stephen Grand Israel National Center for Personalized Medicine (G‐INCPM) Weizmann Institute of Science Rehovot Israel

**Keywords:** cellular senescence, HtrA2/Omi, radiation therapy, siRNA functional screen, vimentin

## Abstract

Radiation therapy can induce cellular senescence in cancer cells, leading to short‐term tumor growth arrest but increased long‐term recurrence. To better understand the molecular mechanisms involved, we developed a model of radiation‐induced senescence in cultured cancer cells. The irradiated cells exhibited a typical senescent phenotype, including upregulation of p53 and its main target, p21, followed by a sustained reduction in cellular proliferation, changes in cell size and cytoskeleton organization, and senescence‐associated beta‐galactosidase activity. Mass spectrometry‐based proteomic profiling of the senescent cells indicated downregulation of proteins involved in cell cycle progression and DNA repair, and upregulation of proteins associated with malignancy. A functional siRNA screen using a cell death‐related library identified mitochondrial serine protease HtrA2 as being necessary for sustained growth arrest of the senescent cells. In search of direct HtrA2 substrates following radiation, we determined that HtrA2 cleaves the intermediate filament protein vimentin, affecting its cytoplasmic organization. Ectopic expression of active cytosolic HtrA2 resulted in similar changes to vimentin filament assembly. Thus, HtrA2 is involved in the cytoskeletal reorganization that accompanies radiation‐induced senescence and the continuous maintenance of proliferation arrest.

## Graphical abstract

Htra2, a mitochondrial serine protease that is a known regulator of apoptosis, was identified through an siRNA screen as a positive mediator of radiation‐induced senescence in cancer cells. Following irradiation, Htra2 enables continuous maintenance of proliferation arrest and cleaves the intermediate filament protein vimentin, leading to filament disassembly and cytoskeletal reorganization.

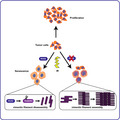

**Abbreviations**
BrdU: bromodeoxyuridine
CTG: CellTiter‐Glo
DAPI: 4′,6‐diamidino‐2‐phenylindole
FDR: false discovery rate
FITC: fluorescein isothiocyanate
GO: gene ontology
IF: intermediate filament
KD: knock‐down
MS: mass spectrometry
PCD: programmed cell death
PI: propidium iodide
SASP: senescence‐associated secretory phenotype
SA‐β‐Gal: senescence‐associated β‐galactosidase activity

## 1 Introduction

Radiation therapy is often used to induce cytotoxic responses such as apoptosis in tumors, particularly in patients with nonmetastatic lung cancer [1], but can also lead to survival responses that avoid cell death, such as autophagy and cellular senescence [2]. Senescent cells are proliferation arrested, and their cytostatic nature precludes further tumor growth. Yet, it is now recognized that at times, growth arrest is reversible, and the dormant senescent tumor cells can regain their proliferative capacity, eventually leading to tumor recurrence [3, 4]. In addition, with time senescent cells develop the senescence‐associated secretory phenotype (SASP), which can lead to the development of a pro‐inflammatory, immunosuppressive microenvironment that favors tumorigenesis [5]. In fact, the presence of senescence markers in patients receiving chemoradiation therapy has been associated with poor prognosis [6]. On the other hand, combining conventional treatment with anti‐senolytic drugs, such as those that inhibit Bcl2 family members, can block tumor progression [7, 8].

Cellular senescence can be a natural consequence of aging, telomere shortening, and replication‐dependent DNA damage (i.e., replicative senescence), or of cell stress induced by certain oncogenes, chemotherapeutic drugs or radiation [5]. Although their proliferation is arrested, senescent cells remain metabolically active and have distinct morphologic and biochemical profiles. Their characteristic features include enlarged area, flattened morphology with a prominent nucleus, accumulation of heterochromatin foci, and enlargement of the lysosomal fraction including enhanced β‐galactosidase activity (known as senescence‐associated β‐Gal, SA‐β‐Gal) [9]. At the molecular level, senescence results from elevated expression of cyclin‐dependent kinase inhibitors such as p21 (*CDKN1A*) and p16 (*CDKN2A*) [9]. This leads to suppression of E2F transcription factors that drive expression of genes involved in DNA replication and the G1/S and G2/M transitions [10]. DNA damage‐induced p53 activation and subsequent increased transcriptional activity is a main regulator of p21 expression.

In order to better understand the contribution of therapy‐induced senescence to the suppression of tumor growth on one hand and to progression and tumor relapse on the other, a more thorough elucidation of its molecular basis is mandated, including mechanisms by which tumor cells can overcome proliferation arrest to escape senescence. Recent studies have explored the latter aspect, implicating several factors, including low levels of thrombospondin‐1 and its receptor CD47 [11], increased CDK4 activity leading to upregulation of the EZH2 methylase [12], which promote senescence escape following chemotherapy‐induced senescence, and enhanced expression of hTERT, which characterizes cells that escaped oncogene‐induced senescence [13]. Here, we used a model of radiation‐induced senescence of the non‐small cell lung cancer cell line NCI‐H460, acquired the proteomic signature of these cells by mass spectrometry analysis, and implemented an siRNA functional screen to uncover factors that regulate induction and/or escape from senescence. We thus identified HtrA2, a mitochondrial Ser protease that is released to the cytosol upon cell stress, as an effector driving the re‐organization of the vimentin intermediate filament (IF) network and maintaining persistent growth arrest.

## 2 Materials and methods

### 2.1 Cell culture and reagents

All reagents were purchased from Sigma‐Aldrich (Rehovot, Israel) unless otherwise indicated. NCI‐H460 and HCT116 cells were purchased from NCI (Manassas, VA, USA) as part of the NCI‐60 Anti‐Cancer Cell Line Panel, and routinely tested for mycoplasma. Short tandem repeat profiling (performed by the Genomics Center of Biomedical Core Facility, Technion, Haifa, Israel, using the Promega GenePrint 24 System) confirmed the authenticity of the cell lines. Cells were cultured in RMPI‐1640 medium (Biological Industries, Beit Haemek, Israel), supplemented with 10% fetal bovine serum (GibcoBRL, Qiryat Shemona, Israel), 4 mm glutamine (GibcoBRL), and 100 µg·mL^−1^ penicillin, 0.1 mg·mL^−1^ streptomycin. NCI‐H460 shRNA transfected cells were grown in the presence of puromycin (3 μg·mL^−1^). Cells were treated with the following reagents: DMSO, TRAIL (100 ng·mL^−1^; PeproTech, Rehovot, Israel), Ucf‐101 (20 μmol·L^−1^; Merck), Q‐VD‐OPh (50 µm; Merck, San Diego, CA, USA). For radiation experiments, NCI‐H460 (8 × 10^5^ cells) or HCT116 (4 × 10^6^ cells) cells were irradiated 24 h after plating by X‐rays at 10 or 8 Gy, respectively, in a single fraction, using XRAD 320 (Precision X‐Ray). Control cells were subjected to mock irradiation. When indicated, Ucf‐101 or DMSO was added 3 h prior to irradiation.

### 2.2 Cell viability assays

The number of live cells was determined by Countess cell counter (Thermo Fisher, Qiryat Shemona, Israel) or manually by averaging the number of cells in 4 1.3 × 1.75 mm^2^ fields for each 10 cm plate under light microscopy. PrestoBlue (Invitrogen, Waltham, MA, USA) and luminescence‐based CellTiter‐Glo (CTG; Promega, Madison, WI, USA) assays were performed according to the manufacturers' protocols; fluorescence and luminescence were measured with a microplate fluorescence reader (TECAN, Tecan Trading AG, Männedorf, Switzerland) or a microplate luminometer (TECAN), respectively. For calcein/propidium iodide (PI) assays, 1 µm Calcein AM (Life Technologies, Hertzliya Pituach, Israel) and 1.5 µm PI were added to culture medium 24 h post‐irradiation and live and dead cells were manually counted under light microscopy (Olympus IX73, Tokyo, Japan).

### 2.3 DNA constructs and transfection procedures

GFP was expressed from pEGFP plasmid. pcDNA3‐delta133Omi‐EGFP was a gift from L. Miguel Martins (Addgene plasmid #14124; http://n2t.net/addgene:14124; RRID:Addgene_14124) [14]. For transient transfection of DNA, TransIT‐X2 reagent (Mirus, Madison, WI, USA) was used according to manufacturer's instructions. For transient siRNA transfections, 25 nm siGENOME siRNA pool to HtrA2 (cat# M‐006014‐04; see Table S1 for targeted sequences of duplexes in pools) or siCONTROL nontargeting siRNA #5 (NT5, cat# D‐001210‐05‐05) was mixed with Dharmafect1 (Dharmacon, Abu‐Gosh, Israel) and added to 2 × 10^6^ cells 24 h after plating. For stable shRNA transfections, NCI‐460 cells were infected with lentiviral particles containing pGIPZ vector carrying shRNA targeting HtrA2 (Dharmacon clone ID V3LHS_315866, mature antisense sequence: ATAAGGTCAGTGTTTCTCG), GAPDH shRNA as positive control or nonsilencing shRNA as negative control, according to the manufacturer's protocol (Dharmacon; cat# VGH5526). After continuous selection with puromycin, the clone exhibiting the most prominent decreased expression level of HtrA2 was chosen for further analysis.

### 2.4 Immunostaining and microscopy

Twenty‐four hours post‐plating in 35 mm glass‐bottom microwell plates, NCI‐H460 cells were irradiated, and after 48 h, fixed with 4% paraformaldehyde and 0.1% glutaraldehyde, washed and permeabilized by 0.2% Triton‐X100 (Merck), followed by overnight incubation with chicken anti‐vimentin polyclonal antibody (Abcam, Tel Aviv, Israel; cat# 24525) in blocking solution (8% BSA, 0.1% Triton‐X100) and then Alexa Fluor 647‐conjugated secondary anti‐chicken IgG antibody (Invitrogen). Cells were then stained with phalloidin‐TRITC and DAPI. Images were acquired with an automated inverted microscope (DeltaVision Elite system IX71 with resolve3D embedded imaging software; Applied Precision/GE Healthcare, Issaquah, WA, USA) using a 60×/1.42 oil objective (Olympus). Further image processing was performed using imagej (NIH Imaging Software) [15].

For bright‐field light microscopy imaging, cells were viewed by UPLFLN PH 10×/0.30 and 20×/0.50 objectives on an Olympus IX73 fluorescent microscope equipped with a DP73 camera or an Olympus IX71 fluorescent microscope equipped with a DP70 camera. Images were captured with Olympus cellsens software.

### 2.5 SA‐β‐gal staining and ImageStream X analysis

SA‐β‐gal staining protocol and ImageStream X analysis were previously described [16]. Samples were analyzed by Amnis ImageStream X Mark II (Luminex, Austin, TX, USA), using dedicated image analysis software (ideas 6.2, Seattle, WA, USA). Cells were gated using the Gradient RMS and contrast features, and according to their area (in μm^2^) and aspect ratio (the Minor Axis divided by the Major Axis of the best‐fit ellipse) of the bright‐field image. The percentage of positive SA‐β‐gal stained cells was determined by the bright‐field mean pixel intensity of each cell (lower values correspond to higher staining).

### 2.6 Cell cycle analysis

Twenty‐four hours following irradiation, NCI‐H460 cells were pulsed with 10 µm bromodeoxyuridine (BrdU) for 3 h at 37 °C, and detached and trypsinized adherent cells collected and fixed in 4% PFA. After consecutive incubations in Denaturation Solution (2 N HCl/Triton X‐100 in PBS) and Neutralization Solution (0.1 m Na_2_B_4_O_7_, pH 8.5), cells were incubated with Antibody Solution [PBS, 0.5% Tween 20, 1% BSA, 1 µg anti‐BrdU‐FITC‐conjugated antibody (eBioscience, Modi’in, Israel)] and then resuspended in DAPI solution. Samples were analyzed by ImageStream X Mark II as above. At least 2 × 10^4^ cells were collected from each sample in each biological repeat.

### 2.7 Protein analysis

Cells were lysed in protein lysis buffer (PLB buffer, 10 mm NaPO_4_ pH 7.5, 5 mm EDTA, 100 mm NaCl, 1% Triton X‐100, 1% Na deoxycholate, 0.1% SDS) supplemented with 10 μL·mL^−1^ 0.1 m PMSF and 1% protease and phosphatase inhibitor cocktails. For vimentin analysis, cell pellets were lysed in 2% SDS, 50 mm Tris‐HCl, pH 6.8 or SDS sample buffer (62.5 mm Tris‐HCl, pH 6.8, 2% SDS, 10% glycerol, 0.1 m DTT). Proteins were subjected to standard SDS/PAGE western blotting, with antibodies against vinculin (Sigma; cat# SA‐V9131), cleaved caspase‐3 (Cell Signaling, Rehovot, Israel; cat# cs9664), tubulin (Sigma; cat# T9026), p21 (Santa Cruz, Petach Tikvah, Israel; cat# 6246), GAPDH (EMD Millipore, Rehovot, Israel; cat# MAB374), HtrA2 (Cell Signaling; cat# cs2176), vimentin (Sigma; cat #V6389), p53 PAb421 antibody [17]. Detection was done with either HRP‐conjugated goat anti‐mouse or anti‐rabbit secondary antibodies (Jackson ImmunoResearch, Petach Tikvah, Israel), followed by enhanced chemiluminescence (EZ‐ECL; Biological Industries Israel Beit‐Haemek Ltd., Kibbutz Beit‐Haemek, Israel).

### 2.8 siRNA functional screening

A custom‐made siRNA library targeting PCD genes (Dharmacon, Table S1) was reverse transfected into NCI‐H460 cells plated in two 96‐well plates. Several of the siRNAs were present in both plates and served as internal standards. Nontargeting siRNA NT5 was used as control. Forty‐eight hours after transfection, one set of the siRNA library pair was irradiated, while its pair was left untreated. Cell viability was measured by CellTiter Glo 48 h post‐irradiation. The experiment was performed in duplicate and repeated four times. Each siRNA readout was log transformed, followed by subtraction of the average siNT5 readouts for each plate, respectively (irradiated or untreated). The fold‐change was obtained by dividing mean values of irradiated by untreated for each siRNA. The statistical difference between irradiated and untreated samples was assessed using a paired *t*‐test, followed by FDR correction (false discovery rate, using p.adjust function) comparing four logarithmic values each for untreated cells and irradiated cells. *P* < 0.05 was considered significant. All calculations were performed with r.

### 2.9 Mass spectrometry‐based proteomic analysis

Mass spectrometry (MS) was performed at the G‐INCPM unit, Weizmann Institute. Total protein lysates were prepared from non‐irradiated and irradiated cells, treated with DMSO or Ucf‐101, 48 h post‐irradiation in four biological replicates. Cells were lysed in buffer (5% SDS, 50 mm Tris‐HCl pH 7.4) followed by sonication. Samples were first subjected to in‐solution tryptic digestion using the S‐Trap method (by Protifi). The resulting peptides were fractionated offline using high pH reversed phase chromatography, followed by online nanoflow liquid chromatography (nanoAcquity) coupled to high‐resolution, high mass accuracy mass spectrometry (Thermo Q‐Exactive HF, Thermo Scientific, Waltham, MA, USA). Samples from each fraction were analyzed on the instrument separately, and within each fraction, in a random order in discovery mode. Data processing was performed using maxquant v1.6.6.0 (Max‐Planck‐Institute of Biochemistry, Martinsried, Germany). The Andromeda search engine was used to search the data against the human proteome database, which was appended with common laboratory protein contaminants and fixed modification (cysteine carbamidomethylation), variable modifications (methionine oxidation), and protein N‐terminal acetylation. The quantitative comparisons were calculated using perseus v1.6.0.7 (Max‐Planck‐Institute of Biochemistry). Decoy hits were filtered out, and only proteins that were detected in at least two replicates of at least one experimental group were kept. A difference ratio for each comparison was calculated based on the average measured protein intensity for each experimental group. A difference in protein level was considered significant if all the following conditions were fulfilled: more than one peptide per protein was identified, the difference ratio was > 2 or < 0.5, and a *P*‐value lower than 0.05 was calculated. Gene Ontology analysis of mass spec results was done using Ingenuity Pathway Analysis (ipa) (QIAGEN Inc., https://www.qiagenbioinformatics.com/products/ingenuitypathway‐analysis) [18]. Top canonical pathways were defined as those with (−log(*P*‐value) > 3).

### 2.10 Statistical analysis

Statistical analyses were performed using Excel, graphpad prism 7 (graphpad Software, San Diego, CA, USA), or r. Values of *P* < 0.05 were considered significant. Specific tests applied to each set of experiments are described in figure legends. Sample variance equivalence was determined by *F*‐test, where appropriate.

## 3 Results

### 3.1 Characterization of radiation‐induced senescence in cancer cells

**Fig. 1:**
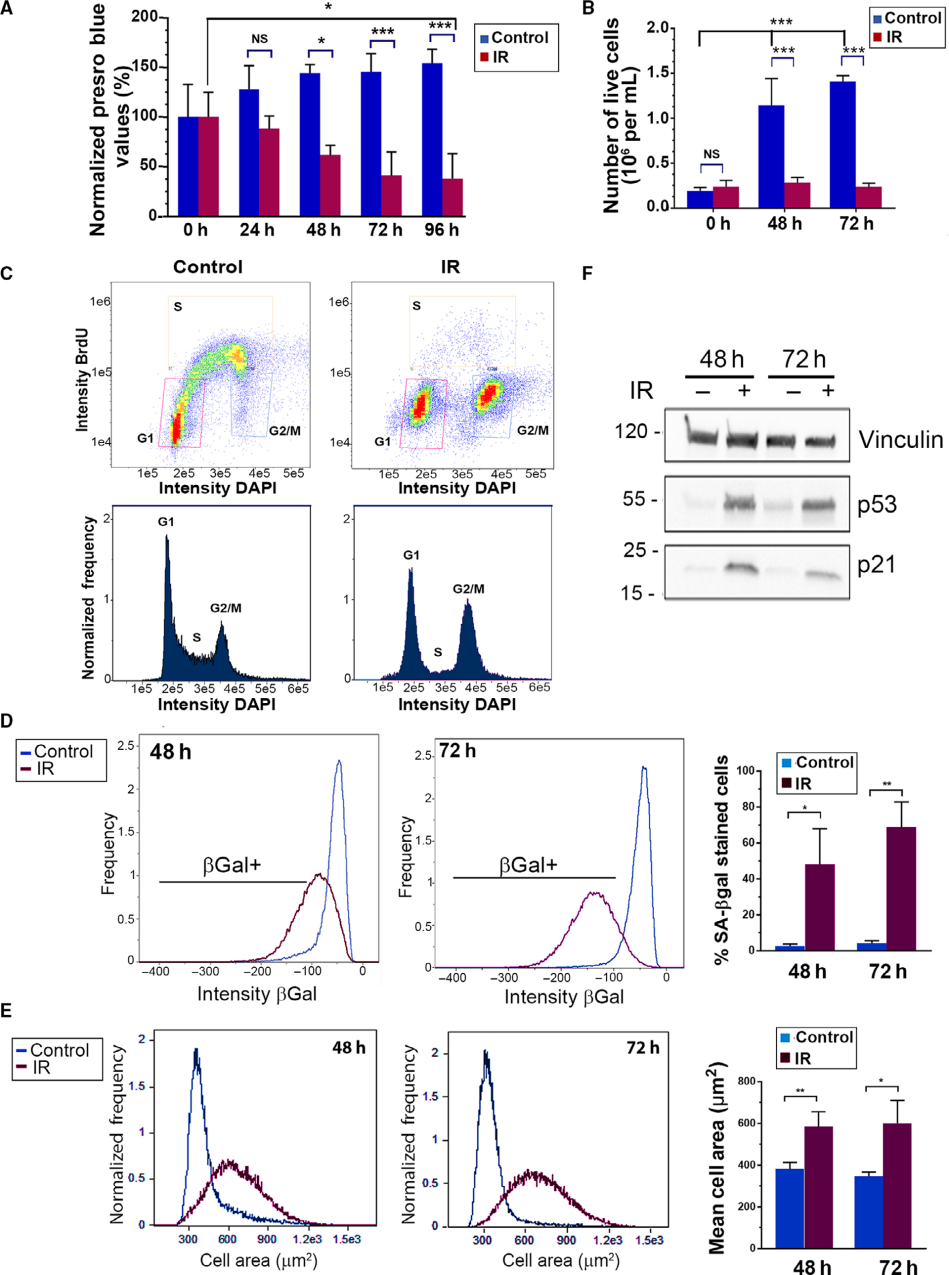
Radiation induces senescence in NCI‐H460 lung cancer cells. (A) Cells were irradiated and cell number/metabolic activity was measured by PrestoBlue assay, which measures viable cells as a function of their reductive activity, at the indicated times post‐treatment. Values were normalized to 0 h. Shown is the mean ± SD of 3 (24 h, 48 h) or 4 (72 h, 96 h) biological repeats per time point. Statistical significance was determined by ANOVA with Tukey's multiple comparison test, *P < 0.05, ***P < 0.0001 (72 h, 96 h), all other comparisons were not statistically significant (ns). (B) Cells were irradiated and live cells counted after the indicated times. Shown is the mean ± SD of 4–6 individual counts from a representative experiment. Statistical significance was determined by ANOVA with Tukey's multiple comparison test, ***P < 0.0001, comparisons across time in irradiated samples were not statistically significant. An additional biological repeat of this experiment can be found in Fig. S1D. (C) Twenty‐four hours post‐irradiation, cells were stained for BrdU and DAPI and analyzed by FACS. At least 2.7 × 10^4^ cells were collected from each sample. Shown is a representative experiment of four biological repetitions. Upper graphs: Dot plots showing BrdU incorporation, population gated by cell cycle stage. Bottom plots: histogram showing DNA distribution, cell cycle stage is indicated. (D, E) 48 or 72 h post‐irradiation, cells were stained for senescence‐associated β‐galactosidase activity (SA‐β‐gal) and DAPI, followed by ImageStream X analysis. At least 2.9 × 10^4^ cells were collected from each sample. Representative distributions indicating intensity of SA‐β‐Gal staining (D) and cell size (E) are shown. Graph on right in D shows the percentage of positively stained cells, as mean ± SD of 5 (48 h) or 4 (72 h) separate experiments. Statistical significance was determined by two‐tailed Student's *t*‐test, *P = 0.00685, **P = 0.00246. Graph in E shows the mean cell area ± SD of 5 (48 h) or 4 (72 h) separate experiments. Statistical significance was determined by two‐tailed Student's *t*‐test, *P = 0.0164, **P = 0.00173. (F) Western blots of lysates from cells 48 or 72 h post‐irradiation. Shown is a representative blot from at least three independent experiments.

As a model for therapy‐induced senescence, NCI‐H460 non‐small cell lung cancer cells, which express *TP53* but not *CDKN2A*, were irradiated with X‐rays at clinically relevant doses (10 Gy). Twenty‐four to forty‐eight hours post‐irradiation, morphologic hallmarks of senescence were observed by phase‐contrast microscopy: most of the irradiated cells were enlarged and flattened, and in contrast to untreated cells, failed to reach confluency (Fig. S1A). This senescent morphology was observed for at least 12 days (Fig. S1B). The number of viable, metabolically active cells, as measured by PrestoBlue assay, started to decline at 24 h compared to untreated cells, and continued to decrease with time (Fig. 1A). This decline was observed as long as 7 days following irradiation (Fig. S1C). Quantitation of live cells indicated that the total number of cells dropped dramatically compared to control untreated cells, which continued to proliferate, but did not significantly change over the 72 h time‐course (Fig. 1B, Fig. S1D). Thus, the large reduction in PrestoBlue values over time most likely does not reflect loss of cells, but rather a decline in their reductive capacity. Staining with calcein/PI to measure live/dead cells at 48 h indicated a low percent dead cells, which could not account for the large decreases in PrestoBlue values and cell number compared to control (Fig. S1A). Furthermore, no caspase‐3 cleavage was evident, and no subG1 DNA was observed, suggesting that apoptosis was not activated in these cells (Fig. S1E, Fig. 1C). Thus, the reduction in cell number compared to control cells most likely reflects a block in proliferation. Cell cycle arrest at the G2 phase was confirmed by FACS analysis for DNA content; at 24 h post‐irradiation, cells accumulated at the G2/M phase at the expense of S phase and did not incorporate BrdU, indicative of a rapid block in cell cycle progression (Fig. 1C). Staining for SA‐β‐Gal and quantification by ImageStreamX flow cytometry indicated an increase in the portion of irradiated cells that were positively stained at 48 and 72 h post‐irradiation, compared to control cells (Fig. 1D, Fig. S1F). Clear differences were also observed when comparing the mean cell areas (Fig. 1E), reflecting the increased cell size following irradiation (Fig. S1F). At the molecular level, protein levels of both p53 and p21 increased post‐irradiation (Fig. 1F). Altogether, these analyses indicate that radiation induces cellular senescence rather than apoptosis in these lung cancer cells. Radiation‐induced senescence was also observed in HCT116 colon cancer cells, which displayed reduced proliferation, enlarged and flattened cell morphology with prominent nuclei persisting for at least 12 days, increased cell size, SA‐β‐Gal accumulation, and induction of p53 and p21 (Fig. S2).

### 3.2 Proteomics profiling of irradiated NCI‐H460 cells

**Fig. 2:**
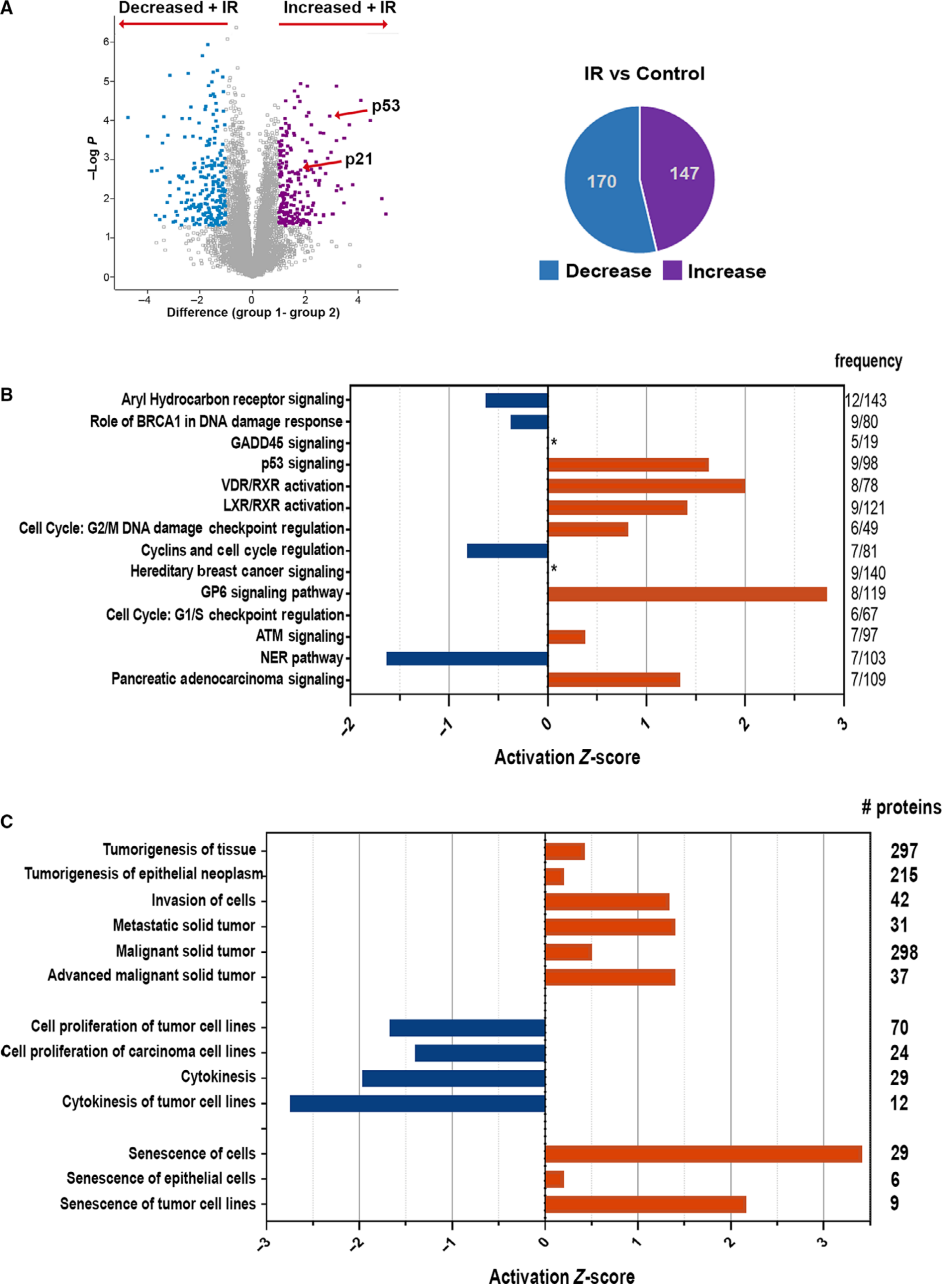
Mass spectrometry analysis of irradiated vs. control non‐irradiated cells. (A) Volcano plot (left) based on protein LFQ‐MS (label‐free quantitation‐mass spectrometry) intensity values. Highlighted data points indicate significantly higher (purple) or lower (turquoise) abundance proteins in irradiated vs control cells (*P* and *q* values < 0.05, fold change > 2 or < 0.5), as summarized in pie chart at right. (B) Ingenuity Pathway Analysis showing the top canonical pathways, listed in descending order, of the dataset of proteins with altered abundance following irradiation. Frequency is defined as the number of proteins in dataset/total number of proteins in the given pathway. Positive and negative *Z*‐scores indicate likely pathway activation and suppression, respectively. *, *Z*‐score undetermined. *Z*‐scores ≥ 2 and ≤ −2 are considered significant. (C) Ingenuity Diseases and Functions analysis of the dataset of proteins with altered abundance following irradiation. Highly significant (*P* < 10^−5^) pathways are shown, grouped by common category. Values on the right refer to the number of proteins in dataset within the given pathway.

In order to characterize alterations in the proteomic profile induced by radiation, mass spectrometry was performed on untreated and irradiated NCI‐H460 cells after 48 h, in order to assess changes that may reflect regulatory processes of the early stages of senescence, to complement recent proteomics studies of the SASP secretome, which catalogues a process developing during later stages of senescence [19]. We assumed that deriving a specific proteomic signature that defines these senescent cells may facilitate further mechanistic understanding of the cellular senescence signaling pathway. If ultimately proven to be similar in other senescence scenarios, the proteomic signature may provide additional molecular biomarkers for monitoring the process, as well as potential therapeutic targets to prevent therapy‐induced senescence in tumors, both of which have been declared important goals of senescence research [20, 21]. A total of ~ 8000 proteins were identified. There was a close correlation within the four biological repeats of each experimental setting (Fig. S3A). Quantitative statistical analysis of irradiated vs. control samples revealed differential expression of 317 proteins (*P* and *q* values < 0.05, fold change > 2 or < 0.5), the abundance of which increased for 170 proteins, and decreased for 147 proteins (Fig. 2A and Table S2). As expected, p53 and p53 response genes were among the proteins with increased abundance (24 total, 14.1% of upregulated proteins), including p21 (CDKN1A), while 107 of the proteins that decreased in abundance (72.8%) were reported targets of p53/p21‐mediated DREAM complex transcriptional repression [22, 23]. Overall, although not excluding translation regulation and post‐translation effects, p53‐dependent transcriptional regulation potentially accounts for 41% of the up‐ and downregulated genes in the dataset, most likely through direct upregulation of *CDKN1A*. Moreover, these results correlate the enhanced p53 and p21 protein levels observed by both western blotting and MS with their functional activation.

The dataset was analyzed by Ingenuity Canonical Pathway and Disease and Function gene ontology (GO) analyses, which provide information as to the likely activation state of a given pathway or function (i.e., a positive *Z*‐score implies a positive correlation with the activated state). As expected, G2/M checkpoint and senescence functions were predicted to be activated, and pathways driving cell cycle progression, cell proliferation, and cytokinesis were predicted to be repressed (Fig. 2B,C). While p53 and ATM signaling are likely activated, the BRCA1‐related DNA damage response is likely to be inactivated (Fig. 2B). Interestingly, malignancy functions featured prominently among the biological processes with positive *Z*‐scores, including pathways such as tumorigenesis, invasion, and metastasis (Fig. 2C). This finding is counter‐intuitive, since it contrasts with the reduced proliferation that was both predicted by the proteomics analysis and observed experimentally (Fig. 1A–C). However, it is in line with recent clinical outcomes of radiation therapy that suggest that in the long‐term, senescence is not a beneficial consequence of cancer therapy and suggests that senescent cells may already be primed toward malignancy from early time points.

### 3.3 siRNA functional screen targeting genes within the programmed cell death pathway identifies HtrA2 as a positive mediator of radiation‐induced senescence

**Fig. 3:**
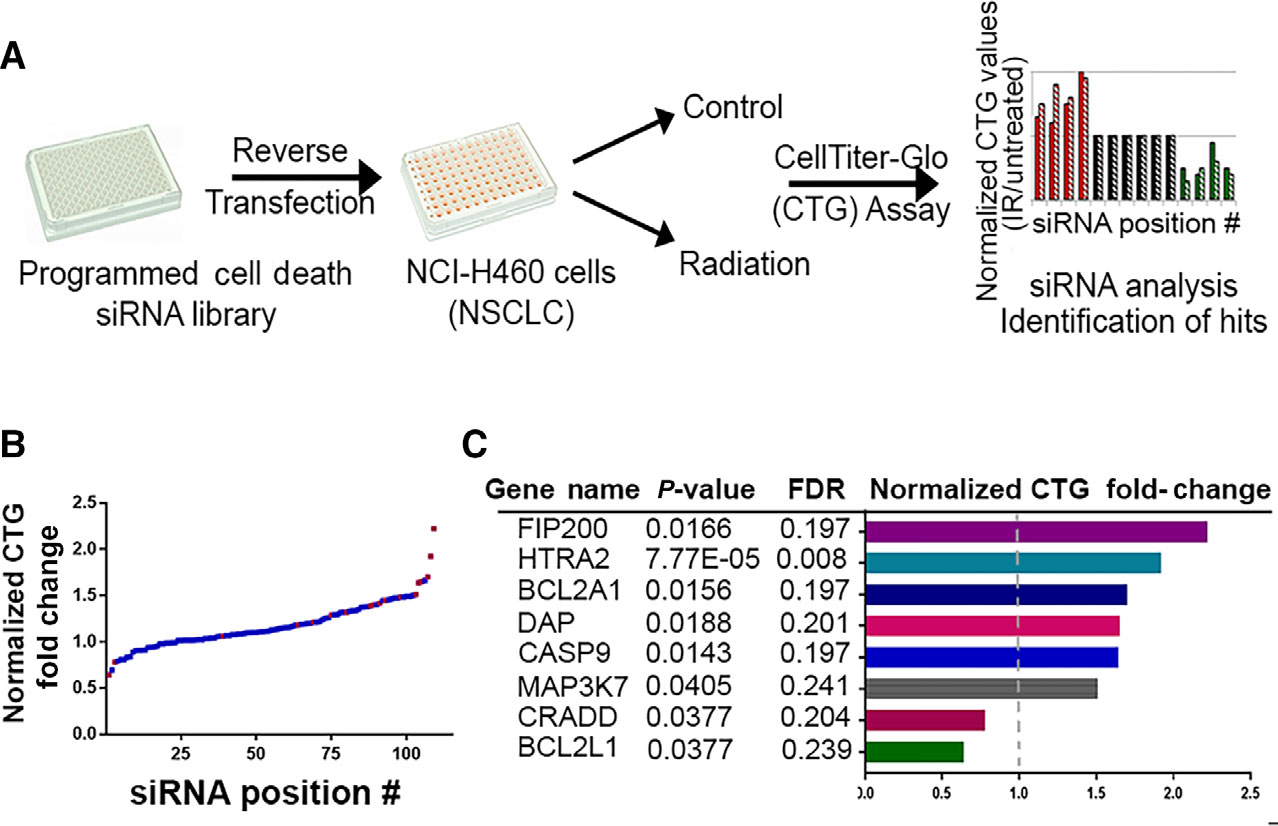
Functional siRNA screen for regulators of radiation‐induced senescence. (A) Schematic of screen using a programmed cell death (PCD) siRNA library to identify gene KDs that either enhance or attenuate the cellular senescent response to radiation, assessed by CTG to quantitate changes in metabolic activity/cell number. (B) Distribution of senescence response of siRNA KDs across the library. For each siRNA, the ratio of CTG values for irradiated vs. untreated cells was calculated. Normalized ratios are plotted for each siRNA, with control nontargeting siRNA values set as 1. siRNAs are distributed along the X‐axis in increasing order of the normalized ratios; some were assayed twice as internal plate controls and both outcomes are plotted. Red points indicate siRNAs with statistically significant fold change, *P* < 0.05. (C) List of genes showing a significant fold‐change in the normalized CTG values. Statistical parameters are indicated, significance was determined by paired Student's *t*‐test. For proteins with increased ratio, only those with a fold‐change > 1.5 are listed.

Having established the senescence phenotype that develops in response to radiation of cancer cells, we next applied a functional siRNA‐based screen for discovering new players that regulate/execute the process. Since a cell's life and death decisions are intricately linked, many points of molecular crosstalk exist within the programmed cell death (PCD) network, and several proteins have dual functions within these various life and death pathways [24]. Senescence and cell death are alternative cell fates that are often triggered by the same stressor, and molecular switches can control whether one or the other is activated [8, 25]. Senescence can thus be considered an annex of the PCD network mediating the balance between life and death of the cell and is likely to share common molecular regulators. For example, p53, p21, and BclX_L_ (BCL2L1) have been shown to modulate both apoptotic cell death and cellular senescence [26, 27, 28]. In order to expand this small sampling and identify additional dual‐function proteins, a small‐scale siRNA‐mediated screen was performed in NCI‐H460 cells. The siRNA library, comprising 97 siRNAs targeting apoptosis, programmed necrosis, and autophagy genes, and also several known regulators of senescence, was applied to both non‐irradiated and irradiated NCI‐H460 cells (Table S1). These were then assayed by CTG, as a measure of metabolic activity/cell number, to identify PCD genes whose knock‐down (KD) affected the senescence response to radiation (Fig. 3A). The screen was performed at 48 h post‐irradiation, to maintain the window of efficacy of the siRNA KDs. Obviously, this screen is limited in that it will not identify non‐PCD regulators that are not included in the siRNA library. A reduction in the response following gene KD (i.e., increased CTG values in irradiated cells) indicates that the corresponding gene functions as a positive mediator of cellular senescence. Since no significant cell death was observed in our model, it is unlikely that increased CTG values upon KD of genes are due to any death‐promoting activities these hits may have. In contrast, enhancement of the response (i.e., decreased CTG values) suggests that either the corresponding gene inhibits senescence, or prevents cell death.

Figure 3B shows the fold‐change in CTG levels, normalized to nontargeting siRNA control, across the PCD library (see Table S3 for raw and normalized values). The change in CTG was statistically significant for 18 genes (Fig. 3B, red points and placed within a map of the PCD network, Fig. S4). The KD of two of these [*BCL2L1* (BCL‐X_L_) and *CRADD*] resulted in an even greater reduction in CTG values compared to control (Fig. 3C, blue circles in Fig. S4), the former consistent with previous reports on the senolytic effect of BCL‐X_L_ KD or inhibition [7, 8, 25, 29]. The KD of the remainder attenuated the response (red circles in Fig. S4); those with a > 1.5‐fold increase in CTG readout are also listed in Fig. 3C. Interestingly, although several pro‐apoptotic genes emerged from the screen (e.g., caspase‐9), the lack of apoptosis in our model indicates that these ‘moonlighting’ genes are playing a different, noncanonical role in cellular senescence unrelated to their apoptosis functions. The top hit that passed the FDR correction threshold was *HtrA2* (Fig. 3C). KD of *HtrA2* resulted in a nearly twofold increase in CTG values following irradiation, normalized to control KD, that is, the radiation‐induced decrease in cell number/metabolic activity was attenuated by depletion of *HtrA2*. This suggests that HtrA2 (also known as Omi, see placement in PCD map, Fig. S4), a mitochondrial Ser protease that is released to the cytosol during cell stress [30], may be a positive mediator of radiation‐induced senescence in NCI‐H460 lung cancer cells.

**Fig. 4:**
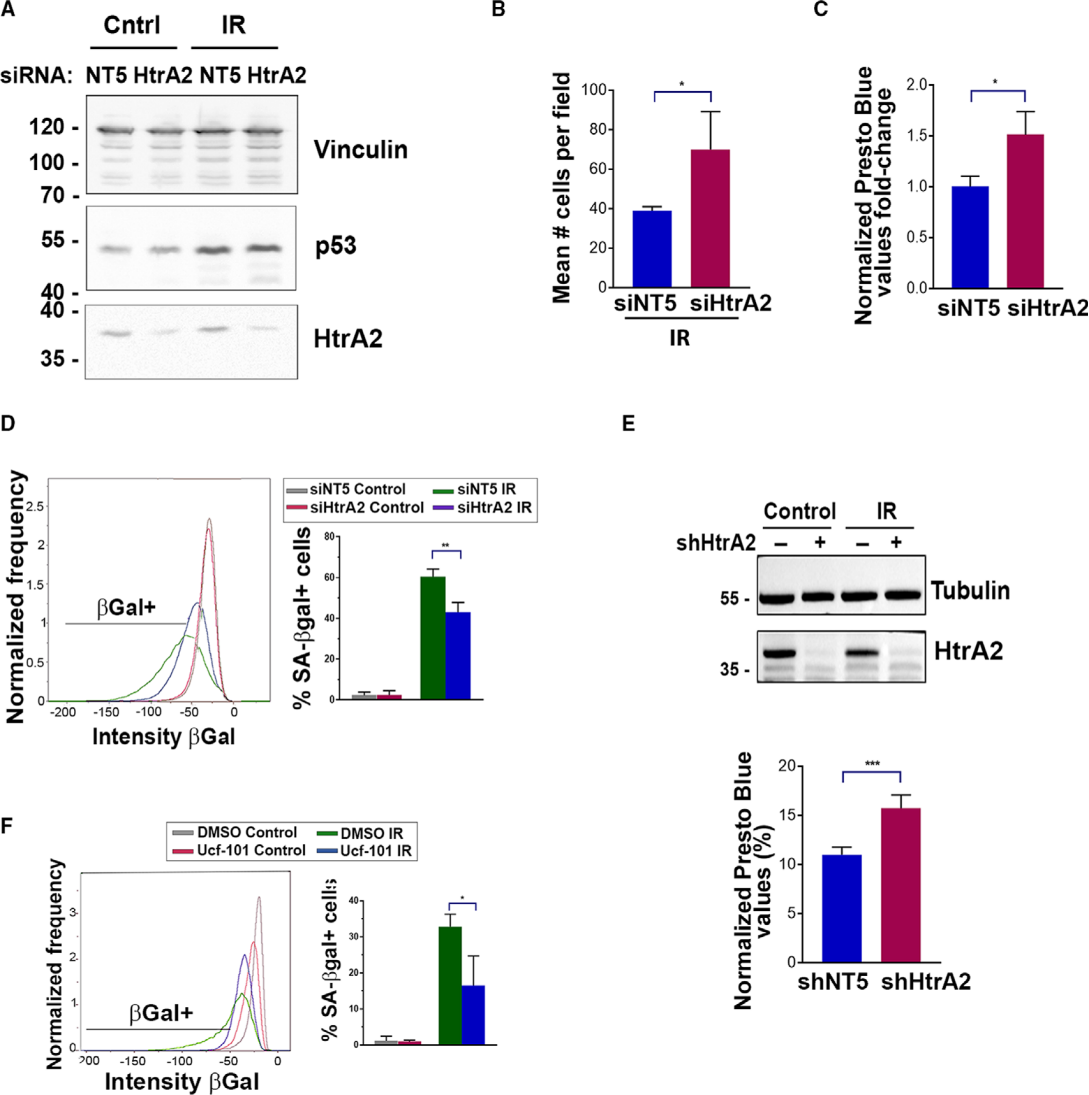
Knock‐down or inhibition of HtrA2 suppresses specific features of radiation‐induced senescence in NCI‐H460 and HCT116 cancer cells. (A) NCI‐H460 cells expressing siRNA to HtrA2 or control siRNA were irradiated and western blotted 48 h later with antibodies to HtrA2 and p53. Vinculin was used as a loading control. Shown is a representative blot from *n* = 4 independent experiments. (B) Cells expressing HtrA2 or control siRNAs were irradiated and the total number of viable cells counted 48 h later. Shown is mean ± SD of three biological repeats, statistical significance determined by Student's two‐tailed *t*‐test, *P = 0.0491. (C) Fold‐change in PrestoBlue values (irradiated/non‐irradiated) 48 h post‐treatment upon KD of HtrA2 by siRNA, with siNT values normalized to 1. Shown is mean ± SD of three biological repeats, statistical significance determined Student's two‐tailed *t*‐test, *P = 0.0235. (D) siHtrA2 or control siNT KD cells were irradiated and stained for SA‐β‐gal and DAPI, followed by ImageStream X analysis after 72 h. At least 1.3 × 10^4^ cells were collected from each sample. A representative distribution is shown, and graph represents mean ± SD of three experiments. Statistical significance was determined by Student's two‐tailed *t*‐test, **P = 0.00743. (E) NCI‐H460 cells stably expressing shRNA to HtrA2 or control nontargeting shRNA were irradiated and 7 days later, subjected to western blotting to confirm KD and PrestoBlue assay to measure viable cell number/metabolic activity. Graph shows mean normalized values (irradiated values divided by control values, expressed as % of control) ± SD of four technical repeats from 1 of 3 representative experiments. Statistical significance was determined by Student's two‐tailed *t*‐test, ***P = 0.000889. (F) HCT116 cells treated with DMSO or Ucf‐101 (20 μmol·L^−1^) were irradiated and stained for SA‐β‐gal and DAPI after 48 h. At least 2.6 × 10^4^ cells were collected from each sample and analyzed by ImageStream X. A representative distribution of SA‐β‐gal positive cells is depicted, and graph represents mean ± SD of three experiments. Statistical significance was determined by Student's two‐tailed *t*‐test, *P = 0.0336.

Western blot analysis confirmed reduced HtrA2 expression upon introduction of siRNA against *HtrA2* in NCI‐H460 cells (Fig. 4A). The size of the HtrA2 band observed (~ 36 kDa) was consistent with the active, soluble form of the protein that results from proteolytic processing of the longer 49 kDa pre‐proenzyme within the mitochondrial intermembrane space after its import to the mitochondria [30]. Consistent with the screen results, the total number of cells (Fig. 4B) and the PrestoBlue values (Fig. 4C) were enhanced by *HtrA2* KD 48 h following irradiation, compared to control KD. KD of *HtrA2* also mitigated the radiation‐induced increase in SA‐β‐gal staining compared to irradiated cells transfected with control siRNA (Fig. 4D), but had no effect on the average increased cell size (Fig. S5A). Consistent with the lack of apoptosis observed, the negligible subG1 DNA content observed by flow cytometry was not affected by HtrA2 inhibition (Fig. S5B). In order to evaluate the contribution of HtrA2 to long‐term senescence, shRNA against *HtrA2* was used to establish stable KD in NCI‐H460 cells. Similar to transient KD cells, the stable *HtrA2* KD cell line exhibited reduced SA‐β‐Gal staining following irradiation, compared to control KD cells, further validating the outcome of *HtrA2* depletion by a different KD strategy targeting a different sequence (Fig. S5C). As observed with transient siRNA transfection, the increased cell size in the irradiated cells was not changed by stable transfection of shRNA to HtrA2 (Fig. S5D). Significantly, while PrestoBlue values remained reduced in irradiated cells even after 7 days, the irradiated stable *HtrA2* KD cells showed higher PrestoBlue values compared to irradiated control KD cells, indicating an increased number of metabolically active, viable cells (Fig. 4E). Finally, to show the relevance of HtrA2 as a regulator of senescence in another cancer cell line, HCT116 cells were treated with an inhibitor of HtrA2 catalytic activity, Ucf‐101, and irradiated. Senescence was measured 48 h later by SA‐β‐gal staining. Significantly, the number of β‐gal‐positive cells decreased in irradiated cells upon HtrA2 inhibition, compared to control‐treated cells (Fig. 4F). This suggests that HtrA2's function in radiation‐induced senescence can be extended to other cancer cell lines.

MS proteomic analysis indicated that overall, the senescence proteomic signature observed after irradiation was only mildly affected by Ucf‐101 treatment; only 51 proteins differed in expression in irradiated cells upon HtrA2 inhibition, 12 of which increased in abundance and 39 decreased (Fig. S3B,C and Table S4). The small size of the Ucf‐101 treatment dataset precluded meaningful GO analysis. Within this dataset, Ucf‐101 treatment produced a reversal of the irradiation‐induced expression changes in seven proteins (Table S4). Significantly, HtrA2 inhibition did not affect the protein expression changes associated with DNA repair, cell cycle arrest, and senescence, pathways identified by the Ingenuity analysis presented in Section 3.2 (Fig. 2B,C). Specifically, p53 and p53‐target genes that were upregulated by irradiation showed no significant change upon HtrA2 inhibition. Likewise, the majority of the 107 p53 repression targets that were reduced in abundance following irradiation was not affected by the addition of the HtrA2 inhibitor (compare Tables S2 and S4). This was not surprising, as p53 and p21 induction was not affected by *HtrA2* depletion or inhibition, respectively (Fig. 4A, Fig. S5E). Thus, although the initial DNA damage response, and p53/p21 functional activation seems to not be dependent on HtrA2, the continuous maintenance of proliferation arrest during senescence does require functional HtrA2, resulting in higher cell number, as well as reduced SA‐β‐Gal activity, upon its depletion.

### 3.4 HtrA2 cleaves vimentin and affects vimentin assembly and polymerization following irradiation

**Fig. 5:**
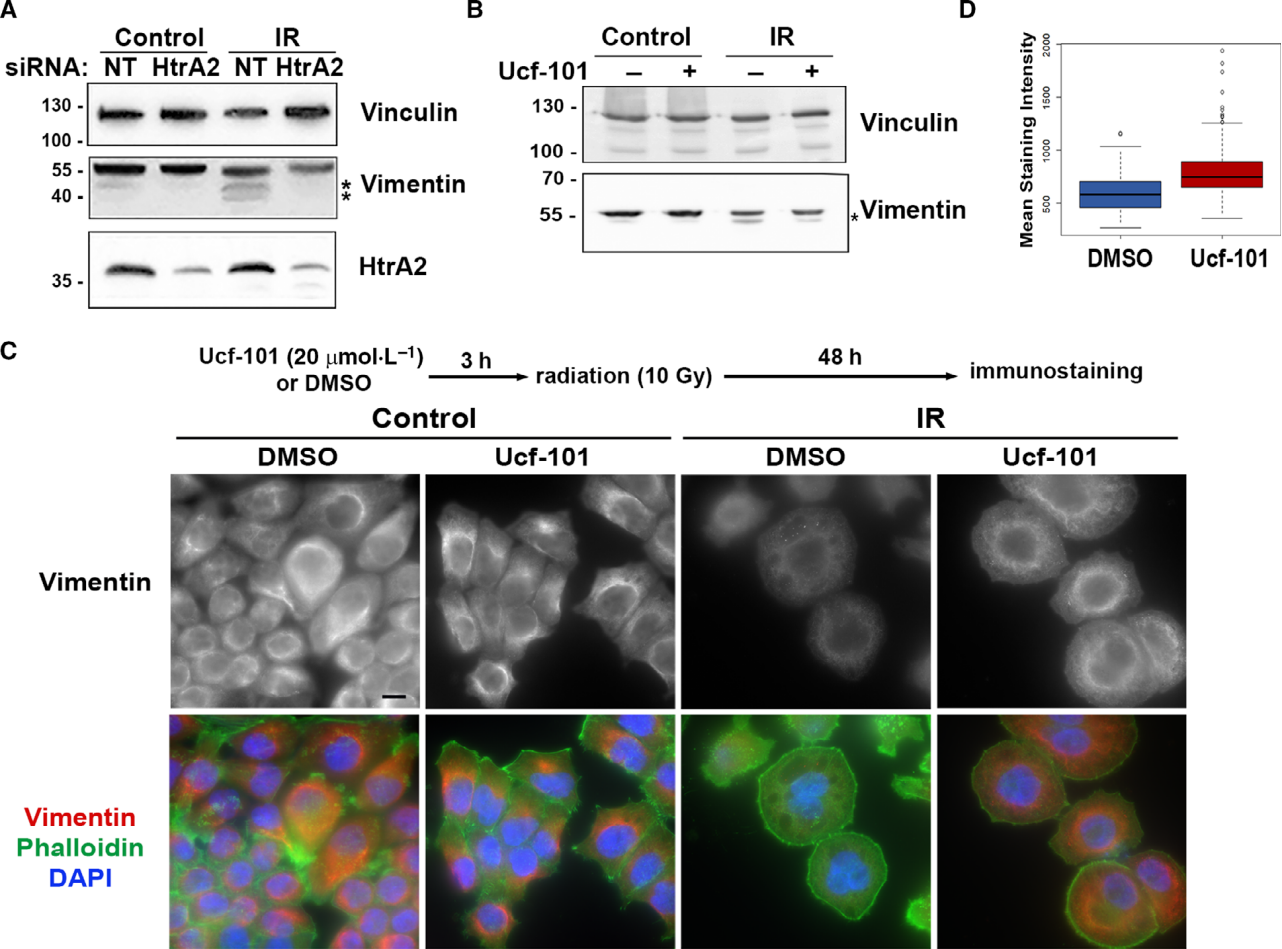
Radiation‐induced cleavage of vimentin and decreases in vimentin staining intensity require HtrA2 proteolytic activity. (A, B) NCI‐H460 cells expressing HtrA2 or control siRNA (A) or treated 3 h prior with either 20 μmol·L^−1^ Ucf‐101 or DMSO as control (B) were irradiated and western blotted 72 h (A) or 48 h (B) later with antibodies to HtrA2 or vimentin. Asterisks indicate vimentin cleavage products. Vinculin was used as a loading control. Shown are representative blots from one experiment done with siRNA, two with shRNA (A), or *n* = 5 experiments (B). (C) Cells treated with either DMSO or 20 μmol·L^−1^ Ucf‐101 3 h prior were irradiated and 48 h later immunostained for vimentin. Phalloidin (green) and DAPI (blue) staining was used to detect actin and nuclei, respectively, and are shown in merged images at bottom. Note that all images are the same magnification, bar 10 μm. Shown are representative images from two independent immunostaining experiments. In experiment 1, 24 and 25 individual multicell fields were viewed for DMSO‐ and Ucf‐101‐treated samples, respectively, and in experiment 2, 25 and 39 fields were viewed for each condition. (D) Box plot showing the range of vimentin staining intensity measured according to the average pixel intensities inside the contours of each cell. Black lines represent median values, colored regions represent the 25th–75th percentiles, and dots represent potential outliers. Statistical significance was determined by two‐sample Kolmogorov–Smirnov test for two biological repetitions, *P* < 2.2e^−16^.

To investigate the p53‐independent function of HtrA2 in senescence, we focused on identifying HtrA2 cytosolic substrates, as previous work demonstrated that it is released from the mitochondria into the cytosol following cell stress [31]. HtrA2 was shown to cleave the IF vimentin *in vitro*, resulting in the removal of part of its N‐terminal head domain, yielding a truncated protein of 49 kDa [32]. Therefore, vimentin expression was assessed here by western blot to determine if it is cleaved in senescent cells. As shown in Fig. 5A,B, a faster migrating form of vimentin was observed at low levels following irradiation in NCI‐H460 cells, consistent with the proteolytically cleaved form of vimentin [33]. The levels of this smaller form of vimentin were reduced in HtrA2 KD cells (Fig. 5A) and upon treatment of cells with Ucf‐101 to inhibit HtrA2 (Fig. 5B, Fig. S5F). Thus, cleavage of vimentin during radiation‐induced senescence requires HtrA2 proteolytic activity.

Previous reports documented that the N‐terminal head domain of vimentin is essential for vimentin filament assembly [34, 35]. To determine if vimentin assembly or localization is affected by senescence in an HtrA2‐dependent manner, immunostaining for endogenous vimentin was performed in irradiated NCI‐H460 cells treated with Ucf‐101, which more uniformly achieves HtrA2 inhibition compared to transient siRNA transfection, which furthermore, does not completely deplete HtrA2 activity (Fig. 5C). These studies were performed 48 h following irradiation, to remain within the window of drug efficacy. In untreated cells, vimentin staining was mostly observed as filaments in a perinuclear ring‐like structure. This pattern was lost following irradiation, and the intensity of vimentin staining was also significantly reduced. Inhibition of HtrA2 activity by Ucf‐101 restored the filamentous staining and blocked the decline in staining intensity, without affecting the overall shape of the senescent cells (Fig. 5C,D). Notably, no obvious changes were observed in the actin cytoskeletal network upon addition of Ucf‐101 within the 48 h time frame post‐irradiation (Fig. 5C).

**Fig. 6:**
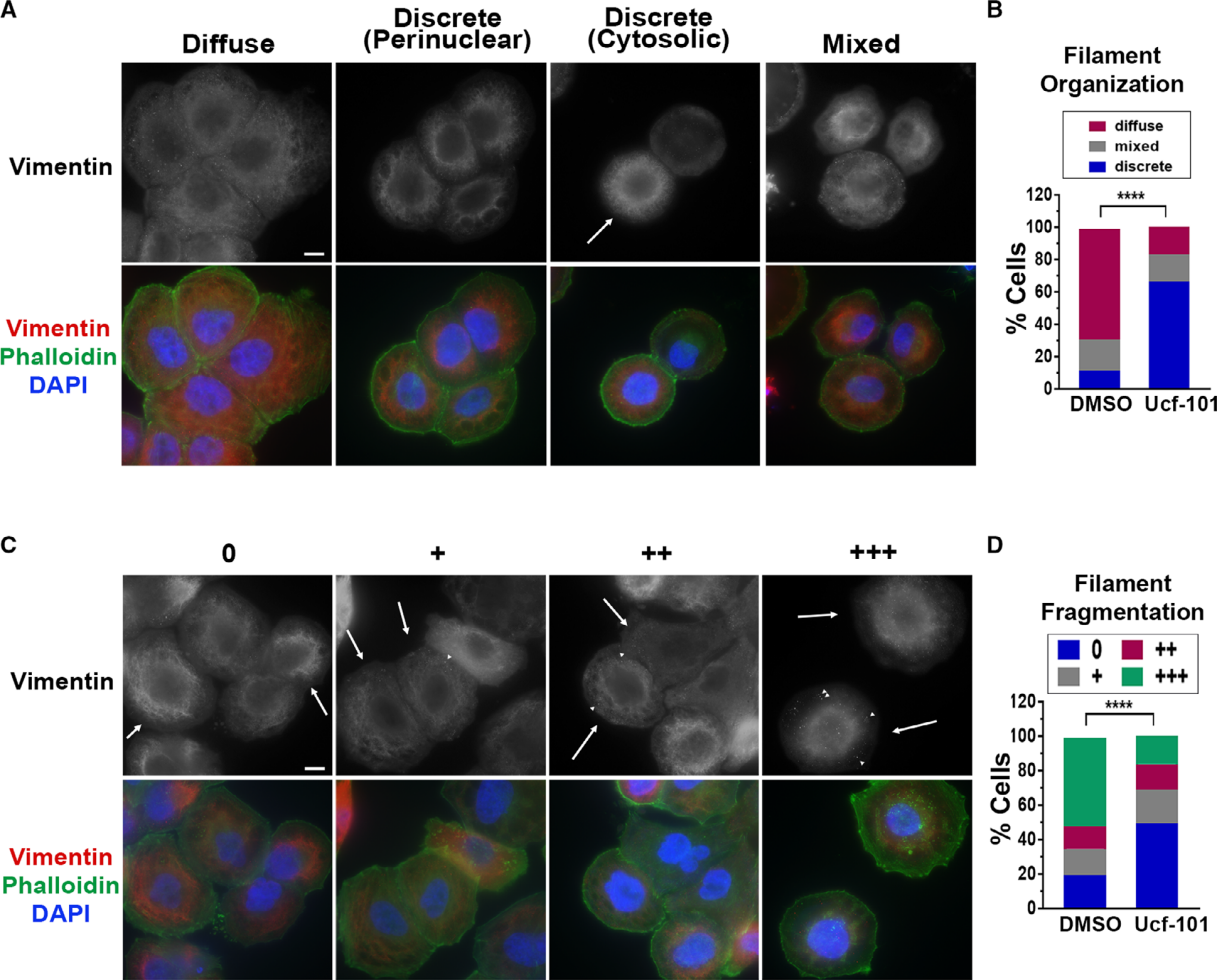
Inhibition of HtrA2 proteolytic activity reduces vimentin filament fragmentation and reorganization following radiation. (A, B) NCI‐H460 cells were irradiated and 48 h later stained for vimentin (red), actin (phalloidin, green) and nuclei (DAPI, blue). Representative images of vimentin‐stained filaments depicting each type of organization are shown in A. Arrows indicate cells displaying the relevant category. Brightness and contrast were adjusted when needed across an image to improve visibility of specific structures. Bar, 10 μm. Quantities of each category in DMSO‐ or Ucf‐101‐treated cells (20 μmol·L^−1^, added 3 h prior to irradiation) are depicted in B. Approximately 200 cells were evaluated in each of two biological experiments. Statistical significance was determined by chi‐square test, ****p < 0.00001. Cytosolic and perinuclear localized discrete filaments were considered as one category. (C, D) Same as A and B, but representing degree of filament fragmentation. Arrows indicate cells displaying the relevant category, arrowheads, vimentin aggregates.

A more comprehensive analysis of vimentin organization in the irradiated cells quantified the changes in vimentin IF assembly and distribution that were mitigated by Ucf‐101. (Note that quantitative comparisons could not be conducted in irradiated vs. control cells, due to the differences in cell size and shape.) Most of the irradiated cells (69%) exhibited diffuse distribution of vimentin throughout the cytoplasm, without visible filaments (Fig. 6A), whereas a minority of cells (11%) displayed discrete, well‐organized filaments in either the perinuclear region or uniformly distributed throughout the cytoplasm, similar to the structures observed in control cells. A fraction of cells (20%) displayed a combination of diffuse and discrete filamentous organization (Fig. 6A), probably representing a transient intermediate stage in the senescence‐related IF disassembly. These changes in vimentin organization were HtrA2 dependent, as the addition of Ucf‐101 to the irradiated cells significantly reduced the fraction of cells displaying the diffuse pattern (17%), so that the fraction of cells exhibiting well‐organized discrete filaments reached 66% (Fig. 6B).

In many irradiated cells that did not exhibit discrete vimentin filaments, globular dot‐like aggregates were detected. These aggregates are presumed to be fragmented vimentin filaments, as described previously [34]. Cells were visually scored for these aggregates as ‘0’ (no visible aggregates), +, ++, and +++ (increasing amounts of vimentin aggregates), as shown in Fig. 6C. Quantitative analysis of these categories indicated that 52% of irradiated DMSO treated cells contained large amounts of vimentin aggregates, with no visible aggregates in only 19%. In contrast, only 16% of irradiated Ucf‐101‐treated cells had large number of aggregates, while 49% did not show any dot‐like aggregates (Fig. 6D).

**Fig. 7:**
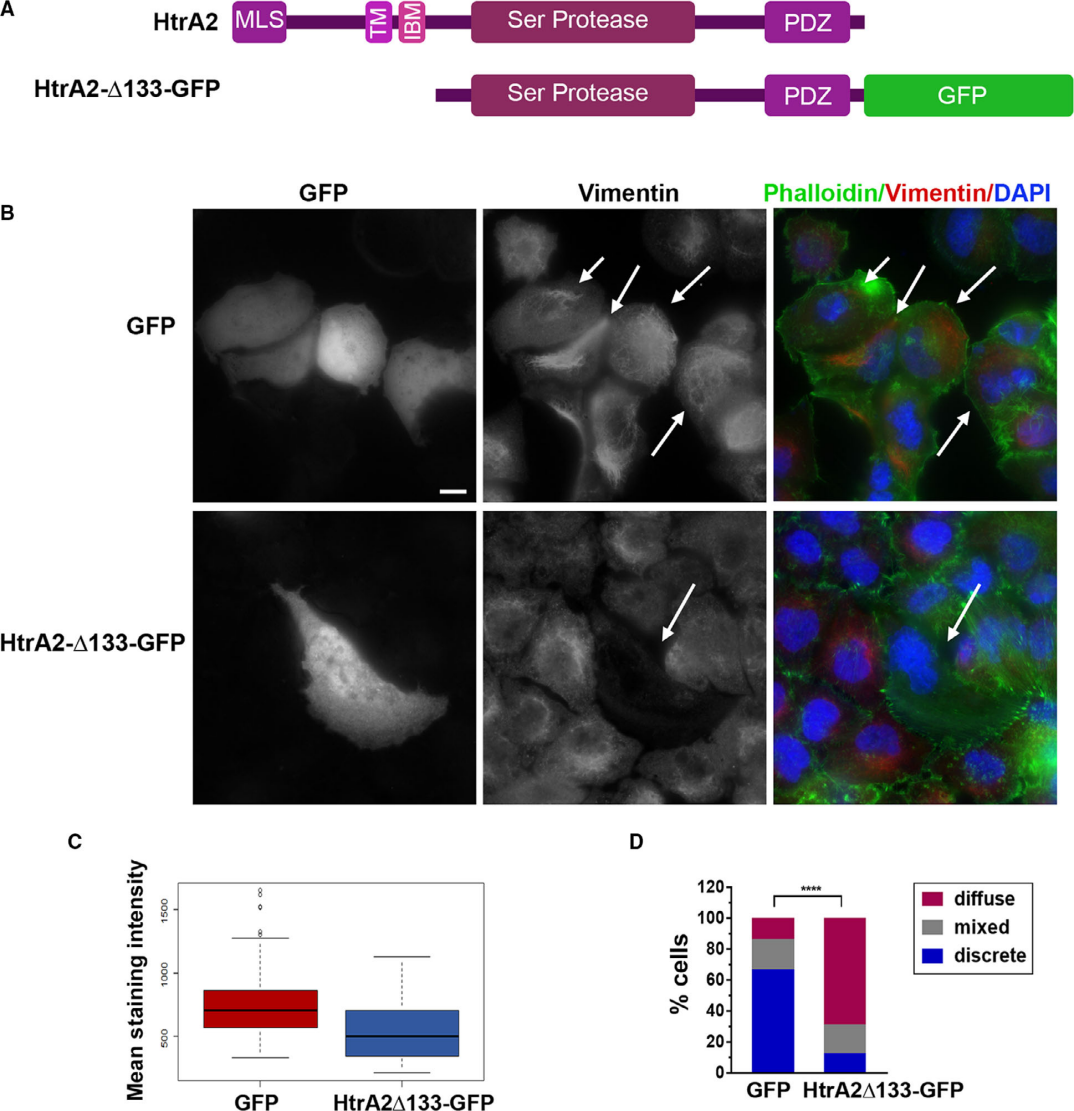
Expression of cytosolic HtrA2 induces changes in vimentin filament organization. (A) Schematic representation of full‐length HtrA2 protein, and the cytosolic HtrA2Δ133‐GFP construct. MLS, mitochondrial localization signal; TM, transmembrane segment; IBM, IAP binding motif; PDZ, C‐terminal PDZ domain. (B–D) Cells treated with Q‐VD‐OPh were transfected with GFP or HtrA2Δ133‐GFP. Forty‐eight hours later, cells were stained for vimentin, actin (Phalloidin, green), and nuclei (DAPI, blue). (B) Representative images of vimentin staining and merged images. Arrows indicate transfected cells. Bar, 10 μm. Staining was performed four separate times with at least 15 fields of cells viewed per experiment, with similar results. (C) Box plot showing the range of vimentin staining intensity measured according to the average pixel intensities inside the contours of each cell. Black lines represent median values, colored regions represent the 25th–75th percentiles, and dots represent potential outliers. Statistical significance was determined by two‐sample Kolmogorov–Smirnov test for two biological repetitions, *P = *1.184e^−06^. (D) Quantification of vimentin filament organization patterns. Statistical significance was determined by chi‐square test for two biological repetitions, ****P < 0.00001.

To test whether the above‐mentioned changes in vimentin filament organization is specifically driven by HtrA2's presence in the cytosol, NCI‐H460 cells were transfected with a plasmid driving the expression of a mature, active cytosolic HtrA2 lacking the mitochondrial targeting signal and tagged at the C terminus with GFP (HtrA2∆133‐GFP) (Fig. 7A). Transfection with free GFP was used as a control. To avoid apoptosis due to expression of large quantities of cytosolic HtrA2, the pan‐caspase inhibitor Q‐VD‐OPh was added. As expected, HtrA2∆133‐GFP localized to the cytosol in non‐irradiated cells (Fig. 7B). The average vimentin staining intensity was significantly lower in the presence of HtrA2∆133‐GFP, compared to GFP control (Fig. 7B,C). Furthermore, the majority of HtrA2∆133‐GFP‐transfected cells exhibited a diffuse distribution of vimentin filaments (70% vs. 14% of GFP‐transfected cells), with only 8% exhibiting the organized discrete filaments, compared to 65% of GFP‐expressing cells (Fig. 7B,D). Notably, phalloidin staining of HtrA2∆133‐GFP‐transfected cells indicated that HtrA2 had no obvious effects on the actin cytoskeletal network (Fig. 7B). Thus ectopic expression of cytosolic active HtrA2 results in similar changes to vimentin assembly as induced by radiation.

In summary, this analysis indicated that following irradiation, concomitant with the acquisition of a senescent state, HtrA2 drives a substantial disruption of the vimentin‐based IF network, characterized by decreased amount of visible filaments and prominent aggregates, consistent with filament fragmentation, and decreased overall vimentin staining intensity. Inhibition of HtrA2's catalytic activity attenuated this process, by significantly preventing the changes in the vimentin filament network. Thus, HtrA2 is responsible for these specific cytoskeletal changes associated with radiation‐induced senescence.

## 4 Discussion

Using a functional siRNA screen of PCD proteins, we identified HtrA2 as a positive mediator of radiation‐induced senescence in NCI‐H460 lung cancer cells, reflecting its ability to maintain sustained proliferation arrest over time. HtrA2 was also shown to be necessary for enhanced SA‐β‐Gal staining, and vimentin cleavage and filament disassembly following irradiation; thus, we conclude that HtrA2 contributes to several hallmarks of the senescence phenotype.

HtrA2 is a Ser protease and chaperone that localizes to the mitochondrial intermembrane space, from where it maintains mitochondrial homeostasis [30]. In this capacity, its inactivation is associated with neurodegenerative disease and aging [36]. During cell stress, however, HtrA2 is released to the cytosol upstream of caspase activation as a result of changes in mitochondria [30]. Within the cytosol, HtrA2 has been shown to induce apoptosis through multiple pathways, including binding to and cleaving IAPs [31, 37]. In the current study, we show for the first time that in addition to its known role in apoptosis, HtrA2 is involved in the regulation of senescence. HtrA2 thus joins the short list of proteins, including p53, p21, and BclX_L_ (BCL2L1), which modulate both apoptotic cell death and cellular senescence [26, 27, 28]. *BCL2L1* also emerged in our screen as a gene whose KD enhanced the radiation‐induced decrease in cell viability. This molecular crosstalk emphasizes the intricacy by which cellular life and death decisions are linked.

As apoptosis is not induced in these cells following irradiation, either additional factors counter HtrA2's ability to cleave its apoptotic substrates (such as BclX_L_ or p21), or the amounts of HtrA2 released to the cytosol are limiting. In either case, HtrA2 is likely to have specific alternative targets/substrates, which require its protease activity for contributing to the senescence phenotype, but which are cleaved independently of caspases [38]. HtrA2 has been shown in the past to cleave several cytoskeletal proteins *in vitro*, including actin and vimentin [32], and to modulate G/F‐actin dynamics in Ras‐transformed senescent fibroblasts by directly cleaving β‐actin [39]. HtrA2 has also been shown to cleave the type III IF vimentin in stressed neuronal cells [33]. While no major changes in the actin filament network were observed following HtrA2 inhibition or overexpression in the current study, HtrA2 proteolytic activity was necessary for vimentin cleavage following radiation in NCI‐H460 cells. This cleavage was previously shown to remove the first 40aa of vimentin, resulting in removal of part of the 95aa N‐terminal head domain [32]. Vimentin's central rod region mediates dimerization and alignment of the dimer into an antiparallel, staggered tetramer, and the exposed head domain interacts with various cellular components, such as lipids, plasma membrane, DNA, and RNA. It is also the site of various post‐translational modifications, including several phosphorylation events, which affect overall filament assembly. Moreover, the most N‐terminal half of the head domain is directly necessary for assembly of the tetramers into filaments [40]. In fact, we show here that filament assembly is disrupted following irradiation, with increased aggregation and diffuse localization. These aggregates most likely correspond to fragmented vimentin filaments, resembling those described in previous work, in which truncated vimentin deleted of the head domain was introduced into vimentin null cells and led to the formation of vimentin aggregates [34]. These changes in vimentin were blocked by inhibition of HtrA2 catalytic activity, and conversely, forced expression of a proteolytically mature form of HtrA2 in the cytosol was sufficient to induce similar changes in vimentin filament distribution and assembly. Although only a small portion of vimentin was cleaved, the functional effect on filament assembly was pronounced. This may suggest that the truncated form acts as a dominant negative to affect assembly of the full‐length protein. While a previous report showed that expression of high levels of human vimentin lacking the entire head in mouse fibroblasts did not affect endogenous vimentin organization [41], other reports are more consistent with the dominant negative hypothesis. For example, some vimentin head mutants and truncations failed to form filaments on their own and disrupted wild‐type endogenous filaments, resulting in the accumulation of aggregates [34]. Interestingly, a mutant variant of vimentin, found in a patient with a premature aging syndrome, promoted the generation of an N‐terminal cleaved form, and faster protein turn‐over [42]. When expressed in vimentin null cells, the mutant failed to from filaments and instead accumulated in cytosolic aggregates, alone or when co‐expressed with wild‐type vimentin. In addition, the cleaved N terminus fragment, although not observed on western blots due to its small size, may remain stable and interact with the acidic central rod domain of intact vimentin. In fact, the head domain was shown to prevent assembly of vimentin into filaments and unravel already formed filaments *in vitro* [43]. Altogether, the data suggest that the direct cleavage of vimentin by HtrA2 within the head domain is a main cause for the loss of vimentin filament organization in irradiation‐induced senescence, although indirect effects of HtrA2 that may influence vimentin assembly by other mechanisms are not excluded.

Although absent in normal epithelial cells, vimentin expression is upregulated during oncogenic transformation [44] and is considered a hallmark of the epithelial–mesenchymal transition (EMT) in motile epithelial cells during embryonic development and in metastatic cancer cells [45]. Changes in vimentin have been previously associated with senescence, although the underlying mechanisms were not explored. For example, doxorubicin‐induced senescence in A549 lung adenocarcinoma cells also led to changes in vimentin filament organization [46]. In adult human fibroblasts, replicative senescence was shown to enhance vimentin expression, and vimentin filament organization differed between aging senescent and young, proliferating fibroblasts [47]. Although the trends in expression and organization differed from that observed in our senescent lung cancer cells, it indicates the significance of vimentin dynamics to different models of senescence. Notably, vimentin IFs are involved not only in defining cell structure and shape but also in cellular motility, organelle positioning, and signal transduction [45]. Moreover, it has been shown to be necessary for tumor growth and proliferation as a result of its ability to act as a scaffold for phospho‐ERK and to promote its phosphorylation [48]. During mitosis, vimentin filaments are found at the cell periphery beneath the actin cortex and are critical for determining the thickness and tension of the actin cortex, which mediates cell rounding [49, 50]. Forced disassembly by HtrA2 during senescence can thus be speculated to be part of the cell's mechanism to prevent cell division, and conversely, restoration of vimentin filament dynamics following HtrA2 inhibition/depletion may allow for cell division once proliferation is restored. Thus, the HtrA2/vimentin axis may control several distinct events during cellular senescence, although additional as yet unidentified HtrA2 substrates may also link it to other molecular pathways mediating the various senescent hallmarks.

To explore such pathways, we compared the proteomic signature of irradiated senescent cancer cells treated with the HtrA2 inhibitor Ucf‐1 to untreated cells by mass spectrometry. The senescence‐associated proteome was only minimally affected by HtrA2 inhibition; it should be noted that post‐translational changes, including direct proteolytic cleavage of potential HtrA2 substrates, were not detected by this method. Yet, the handful of proteins whose radiation‐associated expression changes were rescued by Ucf‐101, and the slightly larger number of proteins whose expression was changed by the inhibitor but not by radiation alone, may provide clues to dissect HtrA2‐mediated pathways. For example, Sumo3, whose abundance decreased upon HtrA2 inhibition in the irradiated cells (Table S4), has been previously linked to senescence, and a Sumo3‐modified proteome was characterized following H‐Ras‐induced senescence in osteosarcoma cells, regulation of which had both pro‐ and anti‐senescent functions [51]. Investigation into the HtrA2 connection to Sumo3 expression may be a worthwhile future endeavor.

## 5 Conclusions

To conclude, we have identified a novel role for the mitochondrial Ser protease HtrA2, previously implicated in apoptosis, in regulating significant cytoskeletal changes occurring during irradiation‐induced senescence, and whose activity maintains the sustained proliferation arrest of senescent cells in association with increased SA‐β‐Gal staining. The increased proliferation observed upon targeting of HtrA2 did not result from attenuation of p53 and p21 expression and their target pathways, implicating other possible mechanisms. We have identified one cell‐based substrate of HtrA2, vimentin; future studies are warranted to determine whether the HtrA2‐vimentin axis is relevant to other models of senescence in cancer cells, and how it may be relevant to primary cells, which do not normally express vimentin, but may induce its expression during senescence. It also remains to be determined whether vimentin cleavage and the consequent filament re‐organization that occurs during radiation‐induced senescence contributes to the sustained proliferation arrest, or whether additional as‐of‐yet unidentified HtrA2 targets are responsible for this function. Escape from senescence is an important factor in limiting the efficacy of tumor treatment modules, including radiation and chemotherapy. Therefore, identification of the relevant HtrA2 substrates will be of great benefit to elucidating the mechanism underlying therapy‐induced senescence.

## Conflict of interest

The authors declare no conflict of interest.

## Author contributions

LH, VL‐S, AK conceived and designed the project; LH performed the experiments with the assistance of NY‐S, MW, SW‐K; LH, VL‐S, SB, BG, AK interpreted the results; NPD‐B helped design the PCD siRNA library; BG and ZP provided expertise and reagents; TO performed data analysis of the siRNA screen; AS and YL performed and analyzed the MS; LH, SB prepared figures and wrote the manuscript; all authors reviewed and edited the manuscript; AK supervised the work and acquired funding.

## Supporting information


**Fig. S1.** Radiation induces senescence in NCI‐H460 lung cancer cells.

**Fig. S2.** Radiation induces senescence in HCT116 colon cancer cells.

**Fig. S3.** Mass Spec‐based proteomics analysis of senescent cells.

**Fig. S4.** Pathway analysis of the hits from the siRNA screen.

**Fig. S5.** Stable knock‐down or inhibition of HtrA2 in NCI‐H460 cells mitigates features of senescence.

**Table S1.** The PCD siRNA library.

**Table S2.** Proteins with changed expression levels following irradiation in senescent NCI‐H640 cells.

**Table S3.** Results of PCD siRNA library screen.

**Table S4.** Proteins with changed expression levels following irradiation with or without Ucf‐101 treatment in senescent NCI‐H640 cells.

## Data Availability

The data that support the findings of this study are available in Figs 1, 2, 3, 4, 5, 6, 7 and/or the supplementary material of this article. Mass spectrometry proteomic datasets are openly available in the PRIDE PXD020972 repository (http://www.ebi.ac.uk/pride/archive/projects/PXD020972).
