# Supplementary material for: A new function for the serine protease HtrA2 in controlling radiation‐induced senescence in cancer cells
Source: Mol Oncol. 2022 Feb 16;16(6):1365–83. doi: 10.1002/1878-0261.13187 (PMC8936513; doi:10.1002/1878-0261.13187)
Supplement: Supplementary file 1 — Fig. S1. Radiation induces senescence in NCI‐H460 lung cancer cells. [file MOL2-16-1365-s006.pdf]

Supplemental Figure S1

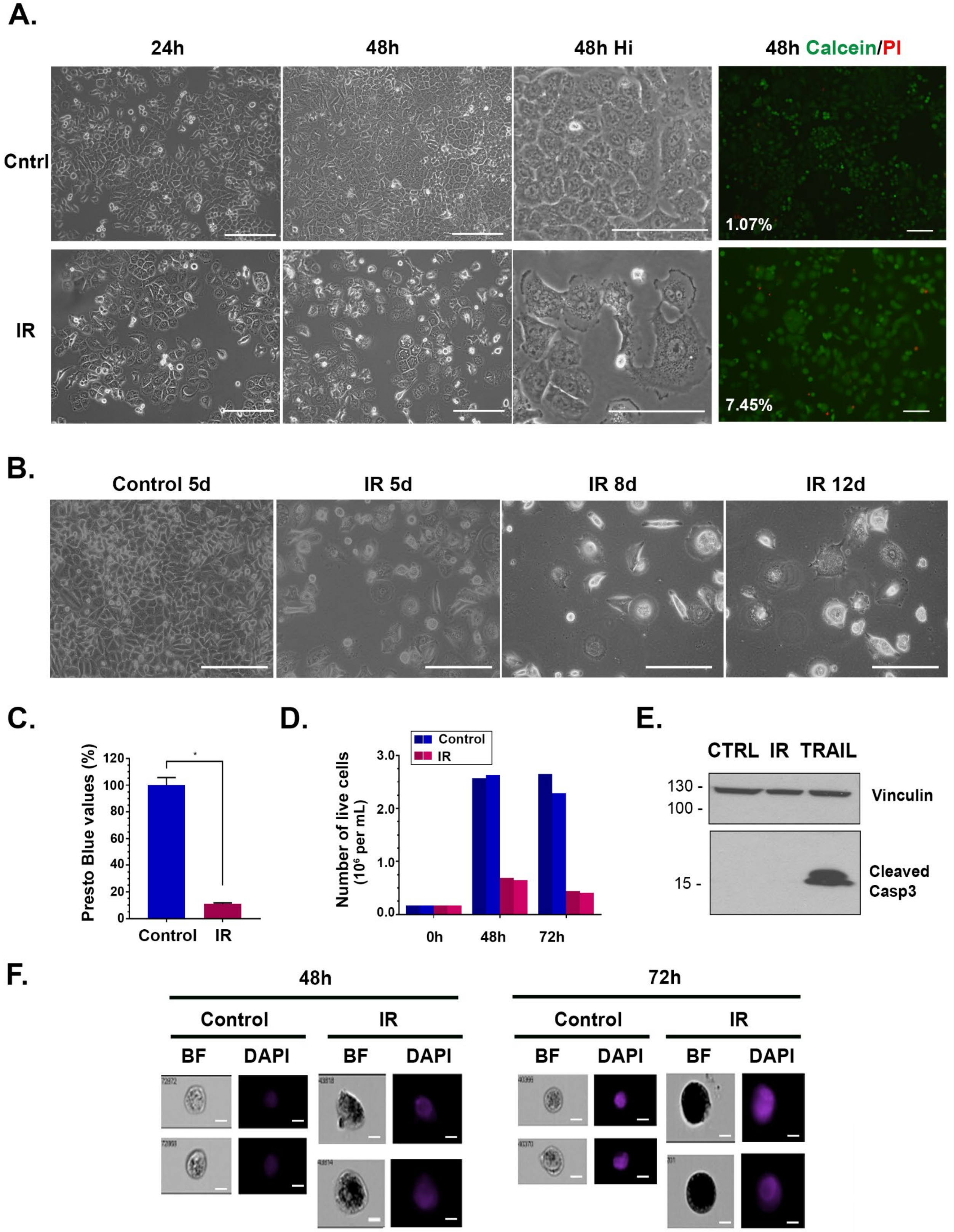

Figure S1. Radiation induces senescence in NCI-H460 lung cancer cells. A. Images of cells viewed by phase contrast light microscopy 24h and 48h post-irradiation. Right panels, 48h post irradiation, cells were stained with calcein (green)/PI (red) and viewed by fluorescent microscopy. The percentages of PI positive cells in control and irradiated cells are indicated. All scale bars are 200 µm, except for higher magnification images at 48h, which are 100 µm. **B.** Images of cells viewed by phase contrast light microscopy 5,8 and 12d post-irradiation. Control non-irradiated cells reached confluence and could not be maintained in culture beyond 5d. Note that scale bars are the same in all panels, 200 µm. **C.** Cells were irradiated or left untreated and PrestoBlue assay was performed 7d later to measure the number of viable, metabolically active cells. Values correspond to the means of n=3 biological repeats, derived from the non-normalized shNT5 controls as shown in Fig 4F. Statistical significance was determined by Student's two tailed T-test, ****, $p<0.0001$. **D.** Cells were irradiated or left untreated and live cells counted after the indicated times, in duplicate. **E.** Cells were irradiated and after 48h, western blotted for the indicated proteins. Lysates from cells treated with TRAIL for 2h were used as a positive control for apoptosis induction. **F.** Representative images of irradiated senescent and untreated cells viewed under brightfield (BF) or fluorescence microscopy by ImageStreamX for DAPI stained nuclei, scale bar represents 10µm in all images.
