# Supplementary material for: A new function for the serine protease HtrA2 in controlling radiation‐induced senescence in cancer cells
Source: Mol Oncol. 2022 Feb 16;16(6):1365–83. doi: 10.1002/1878-0261.13187 (PMC8936513; doi:10.1002/1878-0261.13187)
Supplement: Supplementary file 2 — Fig. S2. Radiation induces senescence in HCT116 colon cancer cells. [file MOL2-16-1365-s004.pdf]

Supplemental Figure S2

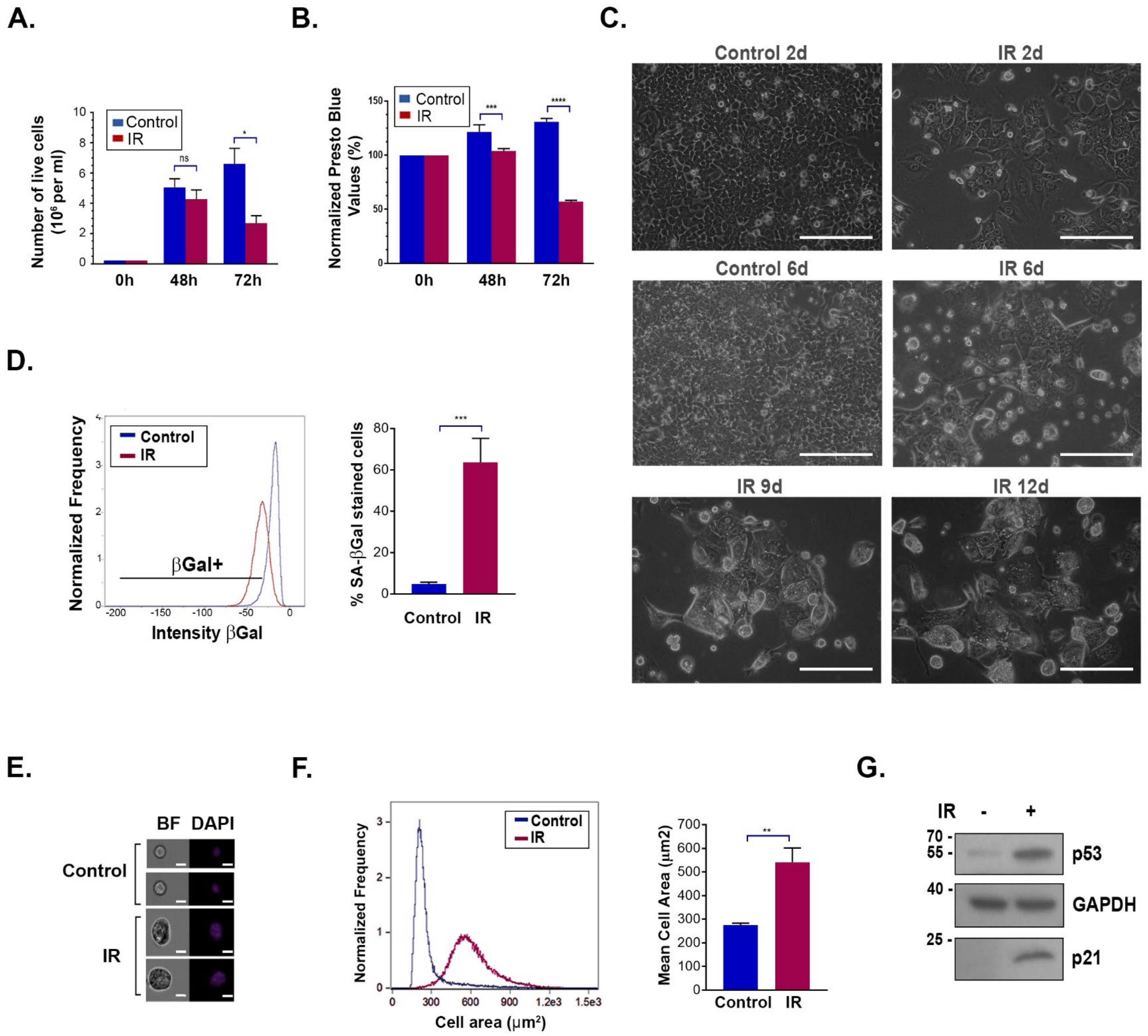

Figure S2. Radiation induces senescence in HCT116 colon cancer cells. **A.** Cells were irradiated and live cells counted after the indicated time. Shown is the mean±SD of two technical repeats from a representative experiment. Statistical significance was determined by Student's two tailed T-test, * p = 0.0391. **B.** Cells were irradiated and cell metabolic activity/number was measured by PrestoBlue assay after the indicated times. Values were normalized to 0h. Shown is the mean of 6 technical repeats ±SD from a representative experiment. Statistical significance was determined by Student's two tailed T-test, *** p = 0.000755, **** $p = 7.46\text{x}10^{-10}$. **C.** Images of

cells viewed by phase contrast light microscopy from 2-12d post-irradiation. Control non-irradiated cells reached confluence and could not be maintained in culture beyond 6d. Note that scale bars are the same in all panels, 200 μm. **D.-F.** 72h post-irradiation, cells were stained for SA-β-gal and DAPI, followed by ImageStreamX analysis. At least 2.1×10^4 cells were collected from each sample. Representative distributions indicating intensity of SA-βGal staining (D) and cell size (F) are shown, graphs on right show the percentage of cells as mean±SD of 3 biological repeats. Statistical significance was determined by Student's t-test, ** $p = 0.00165$, *** $p = 0.00092$. Representative images of irradiated senescent and control untreated cells are shown in E. Scale bar is 10μm in all images. **F.** Western blots of lysates from cells 72h post-irradiation showing p53 and p21 induction. GAPDH was used as a loading control.
