# Supplementary material for: A new function for the serine protease HtrA2 in controlling radiation‐induced senescence in cancer cells
Source: Mol Oncol. 2022 Feb 16;16(6):1365–83. doi: 10.1002/1878-0261.13187 (PMC8936513; doi:10.1002/1878-0261.13187)
Supplement: Supplementary file 3 — Fig. S3. Mass Spec‐based proteomics analysis of senescent cells. [file MOL2-16-1365-s008.pdf]

Supplemental Figure S3

A.

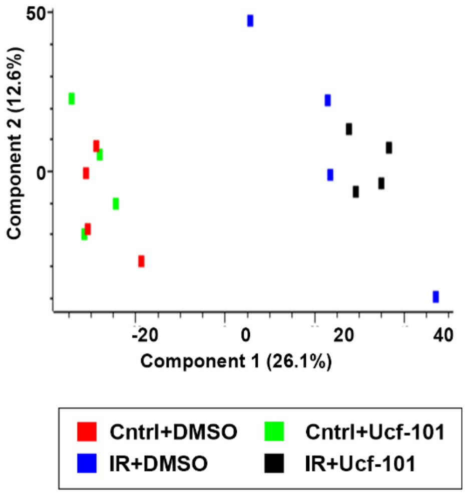

B.

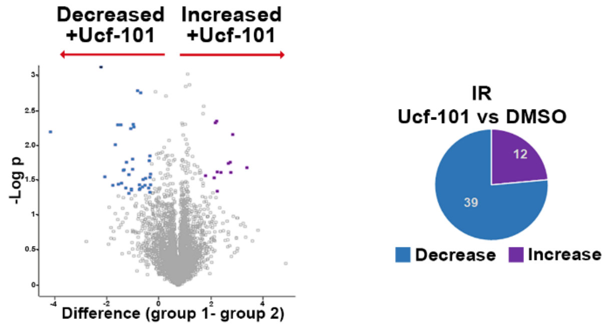

C.

Increased levels +Ucf-101	Decreased levels +Ucf-101
BNIP3L	AGPAT4
AXIN1	NBEAL2
MYH16	MAP2K7
ARG1	FOXJ2
ZNF407	ANKIB1
RGPD5	STK17B
RAD54B	PODXL2
CYP1B1	SIPA1
CAMKK2	QPRT
ZC2HC1A	LIX1L
STEAP2	PRR5L
DHCR7	BRWD1
	NUDT11;NUDT10
	CENPH
	ETV3
	RNF168
	G2E3
	GFOD1
	MMP16
	ROBO1
	SYNJ1
	TMEM132A
	ZNRF2
	HIRIP3
	AEBP2
	RAB39A
	SUMO3
	C19orf25
	ELF1
	CASP2
	MRC2
	HSBP1
	PANX1
	PAIP2
	RASAL1
	BRIP1
	SMIM13
	ZC3H6

Figure S3. Mass Spec-based proteomics analysis of senescent cells. A, PCA analysis showing results of Mass Spec data from control or irradiated NCI-H460 cells, incubated with or without Omi inhibitor Ucf-101 (20 µmol/L), 48h post-irradiation. Each experiment was repeated 4 times. **B.** Irradiated NCI-H460 cells treated with DMSO or Ucf-101 (20 µmol/L) were analyzed by Mass Spec after 48h. Shown is a volcano plot (left) based on protein LFQ-MS intensity values. Highlighted data points indicate significantly higher (purple) or lower (turquoise) abundance proteins in Ucf-101 vs. DMSO treated cells, as summarized in pie chart at right. **C.** Identities of proteins from (B) with increased or decreased abundance.
