# Supplementary material for: A new function for the serine protease HtrA2 in controlling radiation‐induced senescence in cancer cells
Source: Mol Oncol. 2022 Feb 16;16(6):1365–83. doi: 10.1002/1878-0261.13187 (PMC8936513; doi:10.1002/1878-0261.13187)
Supplement: Supplementary file 4 — Fig. S4. Pathway analysis of the hits from the siRNA screen. [file MOL2-16-1365-s005.pdf]

Supplemental Figure S4

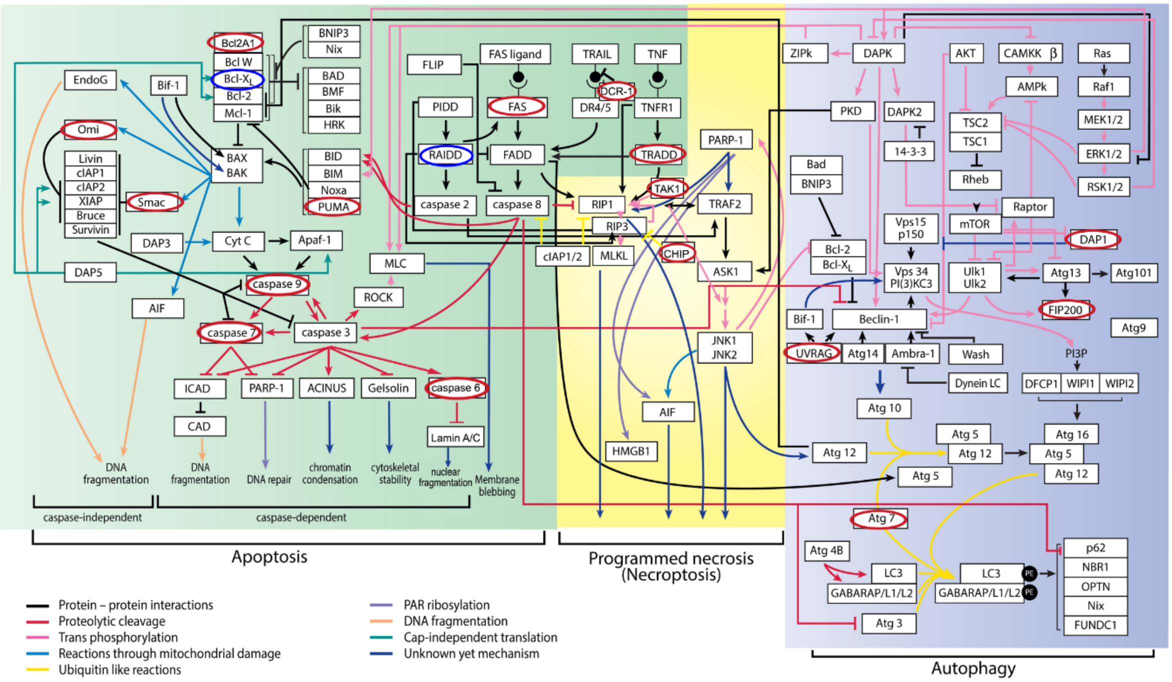

Figure S4. Pathway analysis of the hits from the siRNA screen. A programmed cell death (PCD) map delineating the landscape of protein-protein interactions within the three major modules of PCD, autophagy (highlighted in blue), programmed necrosis (highlighted in yellow) and apoptosis (highlighted in green), based on hundreds of curated publications. The edges possess directionality indicating either activation or inhibition of the target protein and are color coded as indicated. The map was initially published in Ref. 24, and updated herein. Positive hits emerging from the screen whose KD mitigated the decreased viability are indicated by red circles. Negative hits emerging from the screen whose KD enhanced the decreased viability are indicated by blue circles.
