# Supplementary material for: A new function for the serine protease HtrA2 in controlling radiation‐induced senescence in cancer cells
Source: Mol Oncol. 2022 Feb 16;16(6):1365–83. doi: 10.1002/1878-0261.13187 (PMC8936513; doi:10.1002/1878-0261.13187)
Supplement: Supplementary file 5 — Fig. S5. Stable knock‐down or inhibition of HtrA2 in NCI‐H460 cells mitigates features of senescence. [file MOL2-16-1365-s002.pdf]

Supplemental Figure S5

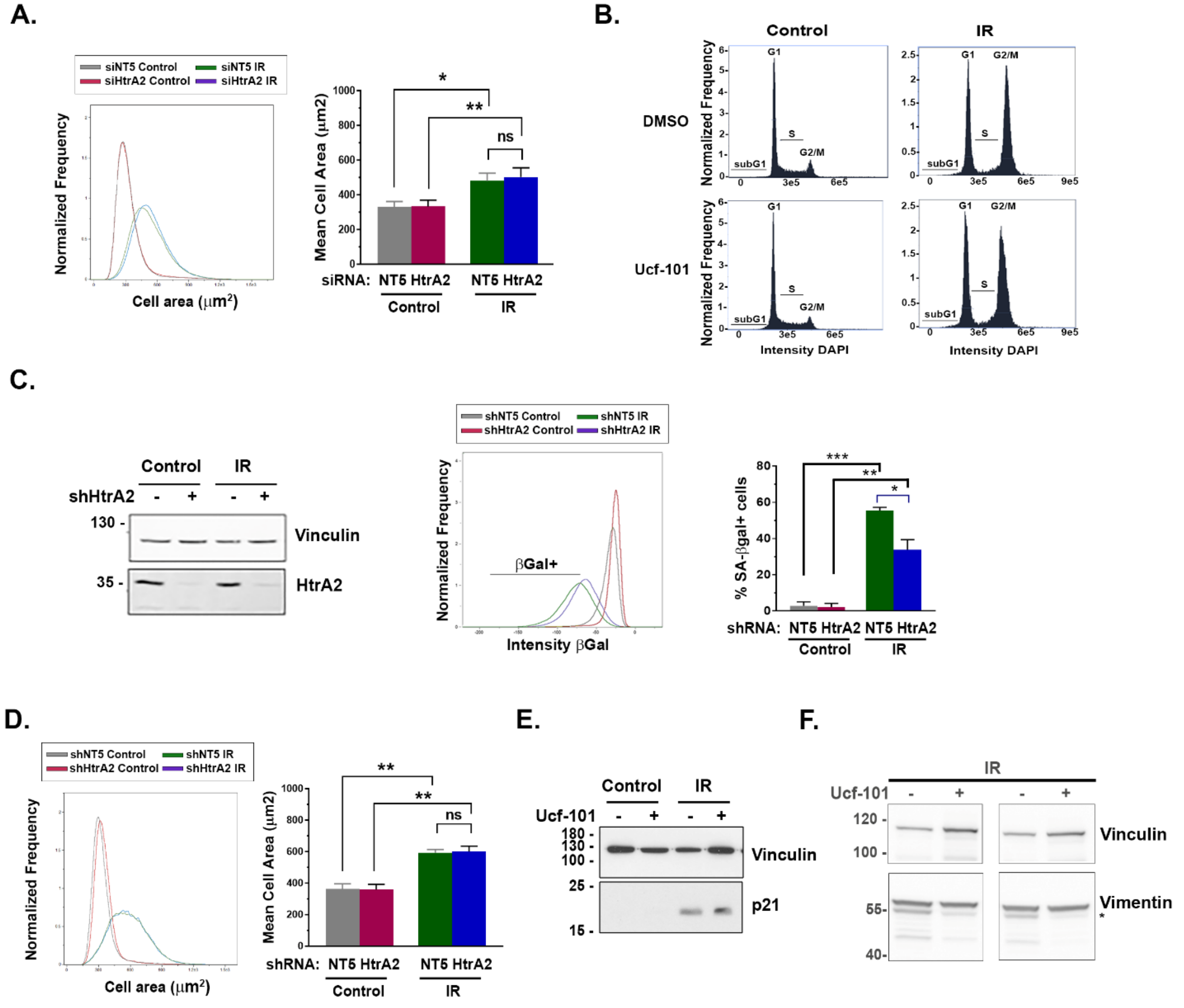

Figure S5. Stable knock-down or inhibition of HtrA2 in NCI-H460 cells mitigates features of senescence. A. NCI-H460 cells transiently transfected with control non-targeting NT5 siRNA or HtrA2 siRNA were irradiated and 72h later stained for DAPI, followed by ImageStream X analysis. Left, representative distributions indicating cell size, right, graph showing the mean cell area±SD of 3 independent experiments. Statistical significance was determined by two tailed T-test, *, p=0.0088, **, p<0.0099, ns, not significant. **B.** 48h post-irradiation, DMSO or Ucf-101 (20 μmol/L) treated cells were stained for DAPI and analyzed by flow cytometry. Shown is a histogram of DNA distribution, cell cycle stage is indicated. Note that the negligible levels of subG1 DNA did not change upon Ucf-101 treatment. **C.-D.** NCI-H460 cells stably expressing shRNA to HtrA2 or control non-targeting shRNA were irradiated

and assayed 48h later. C. Cells were stained for SA-β-gal and DAPI followed by ImageStreamX analysis. At least $3.6x10^4$ cells were collected from each sample. A representative distribution is shown, and graph represents mean±SD of 2 experiments. Statistical significance was determined by Student's two tailed T-test, * * $p = 0.0097$, ** $p = 0.0023$, *** $p = 0.0003$. Western blots of cell lysates at left indicates successful KD of HtrA2. Vinculin was used as a loading control. D. Graph showing the mean cell area±SD as determined from ImageStreamX analysis of 3 independent experiments; a representative distribution is shown at left. At least $3.6x10^4$ cells were collected from each sample. Statistical significance was determined by two tailed T-test, **, $p<0.001$, ns, not significant. **E.** NCI-H460 cells treated with Ucf-101 (20 μmol/L) or DMSO were subjected to western blotting for p21 24h post-irradiation. Vinculin was used as a loading control. **F.** NCI-H460 cells treated 3h prior with either 20 μmol/L Ucf-101 or DMSO as control were irradiated and western blotted 48h later with antibodies to vimentin. Asterisks indicate vimentin cleavage product. Vinculin was used as a loading control.
